# High SOX2 expression is associated with poor survival in patients with newly diagnosed multiple myeloma

**DOI:** 10.1038/s41408-023-00855-1

**Published:** 2023-05-22

**Authors:** Xinhe Shan, Qi Long, Alfred L. Garfall, Sandra P. Susanibar-Adaniya

**Affiliations:** 1 Perelman School of Medicine of the University of Pennsylvania, Philadelphia, PA USA; 2 Department of Biostatistics, Epidemiology and Informatics, Perelman School of Medicine, University of Pennsylvania, Philadelphia, PA USA; 3 Abramson Cancer Center, University of Pennsylvania, Philadelphia, PA USA; 4 Division of Hematology-Oncology, Department of Medicine, Perelman School of Medicine, University of Pennsylvania, Philadelphia, PA USA

**Keywords:** Myeloma, Cancer genomics

Dear Editor,

Relapse is the leading cause of mortality for patients with multiple myeloma (MM). Novel therapies targeting mechanisms of relapse are urgently needed. SOX2 (sex-determining region-box2) is a stem-cell transcription factor whose expression confers drug resistance, enhanced proliferation, and survival advantage to residual myeloma cells, rendering them capable of tumor initiation and survival after cytoreductive therapy [1]. High SOX2 expression is associated with adverse clinical outcomes across several solid malignancies. However, the impact of SOX2 expression in multiple myeloma patients is unknown. We therefore analyzed the association of SOX2 gene expression and clinical outcomes among patients from the CoMMpass study.

The CoMMpass study (NCT145429) is a longitudinal prospective study of 1169 multiple myeloma patients enrolled at the time of diagnosis from 76 sites worldwide that incorporates clinical, genomic, cytogenetic, and transcriptomic data at the time of diagnosis and at each progression event for a minimum of 8 years. The characteristics of patients in the MMRF CoMMpass study are described in the Supplementary Table 1. In this study, we extracted clinical, gene expression (bulk RNAseq on bone marrow aspirate after enrichment for CD138+ cells), and cytogenetic data from Interim Analysis 18 of the MMRF CoMMpass study. Data from the CoMMpass Study was accessed upon request via the MMRF’s Researcher Gateway, an online, open-access portal available at https://research.themmrf.org/.

Among the 1169 patients, 754 have RNA sequencing data of CD138+ bone marrow cells obtained at the time of diagnosis (Supplementary Table 1). SOX2 (ENSG00000181449) expression exhibits a skewed pattern with most patients having low expression (Supplementary Fig. 1). In this analysis, we categorized high SOX2 expression as transcript levels in the top 10% of the whole cohort, and low SOX2 expression as transcript levels in the bottom 90%. We analyzed the association of SOX2 expression with progression-free (PFS) and overall survival (OS). Correlations were drawn between SOX2 expression and myeloma cytogenetic abnormalities of known prognostic significance.

The mean SOX2 expression was 1.146 transcripts per kilobase of exon per million mapped fragments (TPM). The mean SOX2 expression in the high and low SOX2 expression groups were 9.35 and 0.09 TPM, respectively. Table 1 describes the cohort’s demographics, disease characteristics and treatment received stratified according to SOX2 expression. Both groups share similar characteristics with similar distribution of prognostic variables such as ISS staging, deletion 17p (del17p) or t(4;14). Notably, male sex and amplification 1q21(amp1q) were more frequently observed in the low SOX2 expression group. A trend towards higher use of proteasome inhibitors/immunomodulatory agents was seen in the low SOX2 expression group.

**Table 1:** Clinical characteristics according to SOX2 expression in CD138+ plasma cells.

	Low SOX2 expression (*n* = 678)	High SOX2 expression (*n* = 76)	*p*-value
Median age year (range)	64 (27–93)	63 (36–87)	0.56
Gender			**0.01**
Female	266 (39.2%)	40 (52.6%)	
Male	412 (60.8%)	36 (47.4%)	
Race			0.27
White	433 (63.9%)	57 (75%)	
Black	91 (13.4%)	6 (7.9%)	
Asian	11 (1.6%)	1 (1.3%)	
Other/unknown	143 (21.1%)	12 (15.8%)	
ISS stage			0.29
I	232 (34.2%)	26 (34.2%)	
II	246 (36.3%)	23 (30.3%)	
III	181 (26.7%)	27 (35.5%)	
Beta-2 microglobulin	4.9	5.97	0.08
Chromosomal abnormalities			
amp1q	375 (55.3%)	30 (39.5%)	**<0.01**
del17p	62 (9.1%)	5 (6.6%)	0.46
High-risk IgH translocations	100 (14.7%)	7 (9.2%)	0.19
Hyperdiploidy	342 (50.4%)	39 (51.3%)	0.86
First-line treatment			0.05
PI-based	166 (24.5%)	28 (36.8%)	
Combined PI/IMiD-based	474 (69.9%)	43 (56.6%)	
IMIDs-based	36 (5.3%)	5 (6.6%)	
HDCT/ASCT consolidation	364 (53.7%)	35 (46.1%)	0.21
Progressed after 1st line therapy			0.1
Yes	407 (60%)	37 (48.7%)	
No	231 (34.1%)	32 (42.1%)	
Best response = CR	176 (26.0%)	17 (22.4%)	0.64
Cause of death related to MM	113 (57%)	22 (63%)	0.5

*ISS* International Staging System, *PI* proteasome inhibitors, IMiD immunomodulatory drugs, HDCT/ASCT high-dose chemotherapy and autologous stem cell transplantation, *CR* complete response.

Bold values indicates statistical significant at the *p* < 0.05.

Among newly diagnosed myeloma patients, those with high SOX2 expression have worse overall survival (OS) than those with low SOX2 expression (estimated median OS of 57.0 vs. 95.3 months, log-rank *p* = 0.006, Fig. 1a). The median progression-free survival (PFS) at first-line therapy was also shorter in patients with high vs. low SOX2 expression, but this difference was not statistically significant (estimated median PFS of 24.6 months vs. 37.2 months, log-rank *p* = 0.086, Fig. 1b). We performed Cox regression analysis to evaluate the independence of SOX2 expression as a predictor of overall survival. We selected ISS staging and amplification 1q, del17p and known high-risk IgH translocations (t(4;14), t(14;16), and t(14;20)) for the analysis. In the multivariable analysis, SOX2 expression remained a statistically significant predictor of worse overall survival (hazard ratio of 1.6 (1.05–2.3), adjusted *p*-value of 0.028, Fig. 1b). We then used inverse probability weighting (IPW) based on propensity scores to account for other potential confounders such as gender, age, first-line treatment, lines of therapies used and stem-cell transplant status [2]. The predictive power of high SOX2 expression stays significant using the IPW Cox regression model (hazard ratio of 1.7 (1.1–2.7), adjusted *p*-value of 0.019, Supplementary Fig. 2).

**Fig. 1:**
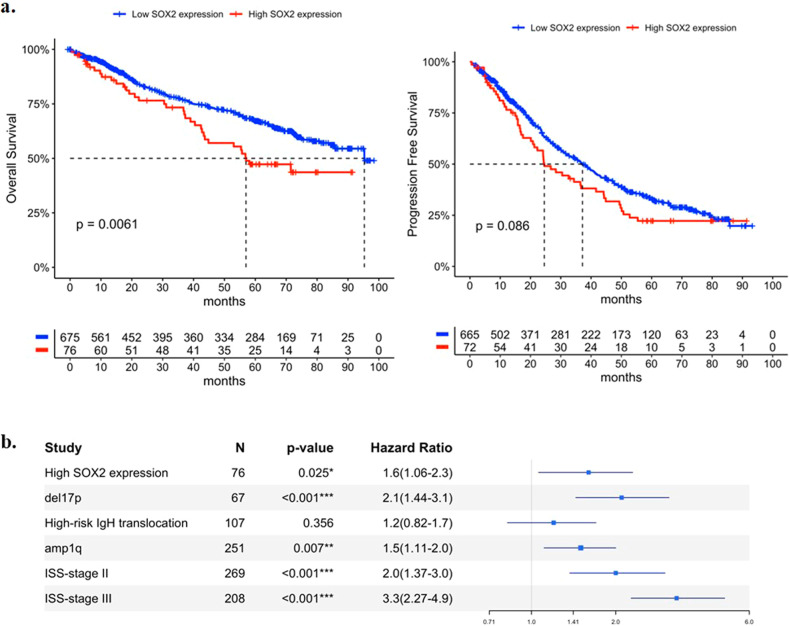
Kaplan–Meier survival curves comparing. **a** Overall survival (left) and progression-free survival (right) according to SOX2 expression in CD138+ plasma cells in patients with NDMM. Censored data is indicated by tick marks. Survival functions were compared using the log-rank test. **b** Cox regression analysis showed that SOX2 expression remains prognostic after adjusting for ISS staging and selected genetic abnormalities.

To investigate the impact of SOX2 expression in relapsed myeloma, we analyzed the outcomes of 377 patients who progressed after first-line therapy. Median overall survival after first progression was significantly worse in the high SOX2 expression group (estimated median OS of 30.0 vs. 52.8 months, log-rank *p* = 0.014, Supplementary Fig. 3a). No significant difference was observed in the time to progression on second-line therapies (estimated median PFS2 of 6.3 months vs. 10.1 months, log-rank *p* = 0.42, Supplementary Fig. 3b).

To further elucidate the biological significance of SOX2 in MM, we applied Differential Gene Expression Analysis using DESeq2 to identify differentially regulated genes in both groups (Supplementary Table 2 and Supplementary Fig. 4). SOX2 overlapping transcript (SOX2OT) was the most upregulated gene in the high SOX2 expression group. SOX2OT has been reported to promote myeloma growth via downregulation of the miR-144-3p/c-MET axis [3]. Another upregulated gene is CCNA1, which encodes cyclin A1, a cancer-testis antigen overexpressed in malignant blasts and leukemic stem cells whose activity is essential for the proliferation of hematopoietic and embryonal stem cells [4]. The most upregulated and downregulated genes underwent KEGG enrichment analysis, which showed that the SOX2 high-expression group is most enriched for gene ontology terms related to cell cycle progression.

Another study using the CoMMpass database showed that EZH2 overexpression correlates with worse prognosis in multiple myeloma [5]. EZH2, a histone methyltransferase, promotes lineage plasticity/pluripotency in prostate [6] and gastric cancer [7] via its regulation of SOX2. Strikingly, the characteristics of the EZH2 overexpression and SOX2 high-expression groups are different. Specifically, EZH2 overexpression is correlated with the presence of amp1q and del17p, whereas we showed that high SOX2 expression is associated with lower likelihood of having amp1q. Furthermore, in the differential gene analysis of stemness genes between high and low SOX2 expression groups (two-time expression, adjusted *p*-value less than 0.05), EZH2 failed to achieve statistical significance (Supplementary Fig. 5). This discrepancy could suggest that SOX2 mediates the progression of multiple myeloma through different mechanisms than EZH2. Further research is needed to uncover the exact role of SOX2 and EZH2 in multiple myeloma.

Our study shows that high SOX2 expression portends worse survival in a large cohort of patients with newly diagnosed MM (NDMM) treated with novel therapies. This association remains significant after adjusting for patient, disease, and treatment characteristics; and is also observed in the early relapse setting. Amplification 1q, a poor prognostic marker in MM, was more prevalent in the low SOX2 expression group; the nature of this relationship is unclear.

Though the definition of high SOX2 expression was set arbitrarily in this study, our data suggest that high SOX2 expression can identify a distinct myeloma subtype enriched for a stem-cell gene signature. A limitation of our study is the use of bulk rather than single-cell RNAseq, which precludes analysis cell-to-cell heterogeneity in SOX2 expression. High Sox2 expression by bulk RNAseq may represent either high global SOX2 expression or a higher proportion of SOX2-high cells, which may also be present in lower frequency in patients with low Sox2 expression by bulk RNAseq. Consistent with this possibility, a recent single-cell RNA-seq identified a cluster of myeloma cells with high SOX2 expression in patients with relapsed refractory MM [8].

Our findings are in-line with other studies supporting the role of cancer stem-cell and lineage plasticity phenotypes in the pathogenesis of multiple myeloma. In pre-clinical models, Sox2 is required for clonogenic myeloma growth and relapse from cytoreductive therapy [1, 9]. Expression of lineage plasticity genes is enriched among residual myeloma cells persisting after CAR T-cell therapy in myeloma patients [10]. In patients with myeloma precursor conditions, T-cell immune responses against SOX2 are associated with lower risk of disease progression to symptomatic MM [11]. Similarly, we identified anti-SOX2 cellular immune responses in MM patients who derived clinical benefits from CAR T-cell therapy [12, 13]. These observations provide rationale for therapeutic approaches that target these stemness phenotypes. In conclusion, we show that SOX2 expression in myeloma cells is associated with worse prognosis in a large cohort of patients with newly diagnosed MM, and its impact was independent of other established prognostic factors including cytogenetics and ISS stage. Further studies are needed to elucidate how SOX2 is regulated in MM and whether novel therapies could target SOX2.

## Supplementary information

Supplementary data and figures version 2

## Data Availability

The data supporting the findings of this study was obtained from Interim Analysis 18 of the MMRF CoMMpass study. These data can be accessed upon request via the MMRF’s Researcher Gateway, at https://research.themmrf.org/.
