# Supplementary material for: High SOX2 expression is associated with poor survival in patients with newly diagnosed multiple myeloma
Source: Blood Cancer J. 2023 May 22;13(1):86. doi: 10.1038/s41408-023-00855-1 (PMC10203273; doi:10.1038/s41408-023-00855-1)
Supplement: Supplementary file 1 — Supplementary data and figures version 2 [file 41408_2023_855_MOESM1_ESM.pdf]

Supplementary data and figures

Supplementary Table 1. Characteristics of the MM CoMMpass database

	**Total patients (n=1143)**
**Median age yr (range)**	63(27-93)
**Gender**	
**Female**	453 (39.6%)
**Male**	690 (60.4%)
**Race**	
**White**	742 (64.9%)
**Black**	161 (14.1%)
**Asian**	18 (1.6%)
**Other/Unknown**	49 (4.3%)
**ISS Stage**	
**I**	401 (35.1%)
**II**	401 (35.1%)
**III**	311 (27.2%)
**Beta-2 Microglobulin**	5.04
**Chromosomal abnormalities**	
**amp1q**	328 (28.7%)
**del17p**	86 (7.5%)
**High-risk IgH translocation**	284 (24.8%)
**Hyperdiploidy**	498 (43.6%)

**First line treatment**	
**PI-based**	272 (23.8%)
**Combined PI/IMID-based**	803 (70.3%)
**IMIDs-based**	68 (5.9%)
**HDCT/ASCT consolidation**	608 (53.2%)
**Progressed after 1st line therapy**	
**Yes**	400 (35.0%)
**No**	669 (58.5%)
**Best Response = CR**	292 (25.5%)

Supplementary Table 2. Top 50 differentially expressed genes between the high SOX2 expression and low SOX2 expression groups

**Gene symbol**	**log2FoldChange**	**Adjusted p value**
SOX2-OT	3.051860568	9.66E-50
CCNA1	4.695017539	1.73E-41
GLP1R	4.827203905	3.36E-36
MTND1P23	-4.172523391	1.03E-34
FUT9	5.205429765	1.31E-33
SHOX2	3.322522941	1.60E-32
TNFSF4	2.352771279	1.28E-30
OTX1	2.703376325	1.03E-28
IGKV1-33	-6.459466452	2.79E-28
AC233755.1	-4.512456373	7.27E-28
IGKV1D-33	-5.949986242	5.84E-27
SYT17	3.312003912	6.51E-27
THEMIS	3.897518365	6.82E-25
IGHV3-30	-4.292928285	2.07E-23
DCLK2	2.851354764	2.73E-22
PRAMENP	3.626322896	6.34E-21
IGHV3-64	-3.990407134	1.11E-20
OVCH1-AS1	-2.775737403	1.53E-20
SDC2	2.85828536	4.88E-19

IGLV3-1	-3.61575613	1.90E-18
IGHV4-28	-3.441769734	8.96E-17
AC020659.1	-2.823241028	1.74E-16
OVOL3	3.117985521	2.42E-16
IGHV1-69D	-3.25140954	1.25E-15
AC019080.1	0.896185459	2.18E-15
IGLV1-41	-3.320484175	2.81E-15
AC002127.2	2.262130886	3.51E-15
IGKV1-39	-3.770854356	3.59E-15
IGHV3-20	-2.968446345	1.19E-14
CYP26A1	2.208825803	1.88E-14
SLITRK2	-5.105130319	4.68E-14
PPP1R14A	-3.587248305	8.78E-14
IGLL5	-2.335017533	1.38E-13
IGKV3-11	-3.179168161	1.92E-13
CDKN2C	1.62806814	2.17E-13
DGKA	0.793175581	5.10E-13
IGHV3-69-1	-3.113541768	7.72E-13
SYN3	2.48607278	8.40E-13
IGHV3-48	-2.812367481	1.50E-12
NEGR1	1.929917429	1.73E-12
IGHV3-33	-3.041780025	1.73E-12
TBC1D3E	-9.884557447	1.77E-12

IGHV3-21	-2.793066437	1.92E-12
IGHV4-34	2.044317048	2.57E-12
SATB2	1.075903815	4.90E-12
IGLV1-44	-3.074951341	5.15E-12
SLC4A10	2.611464058	6.00E-12
RPL3P4	-1.835630199	1.89E-11
IGHV4-55	-2.864980612	2.15E-11
AC139493.2	-4.049163903	4.56E-11

Supplementary Figure 1. Bar plot of SOX2 expression distribution

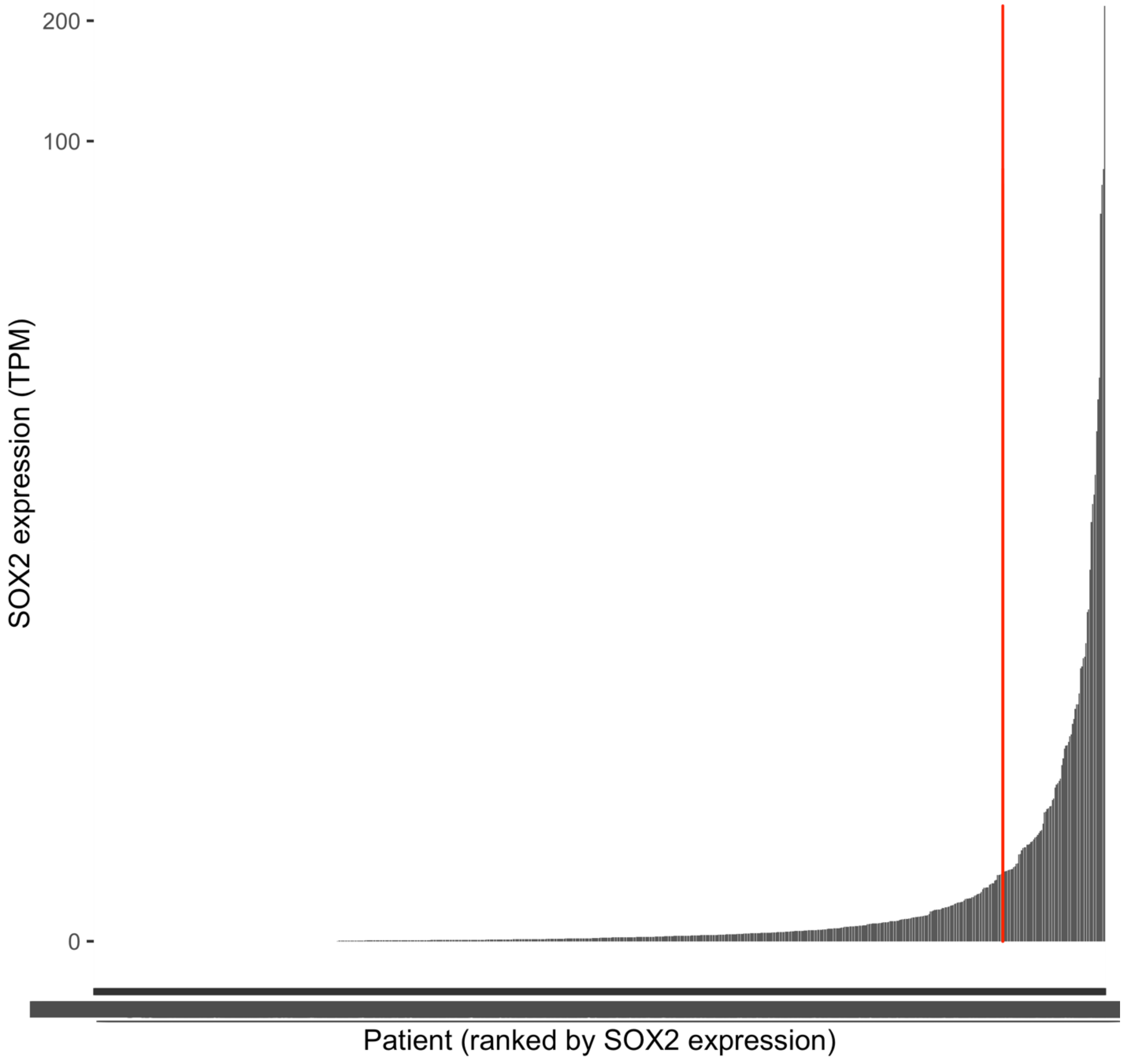

754 patients are ranked in an ascending order on the x-axis. The y-axis is in pseudo-logarithmic scale. The red line denotes the cutoff point for top 10% of SOX2 expression.

Supplementary Figure 2. Forest plot of high SOX2 expression hazard ratio using a weighted Cox regression model

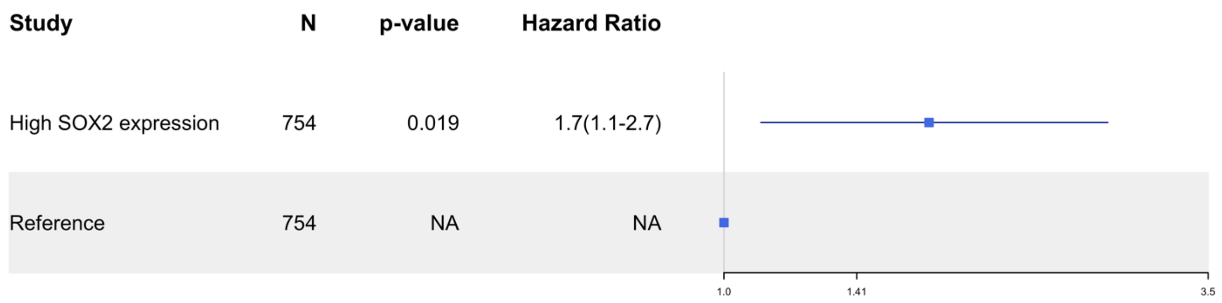

Inverse probability weights were used in the cox regression to estimate the hazard ratio of high SOX2 expression. The inverse probability weights were calculated based on propensity scores that measures how likely someone will be in the high SOX2 expression group based on gender, age, first-line therapy, lines of treatment used and stem cell transplant status.

Supplementary Figure 3. Kaplan-Meier Curves for Overall Survival after first relapse and Progression-free Survival to second line therapy.

a.

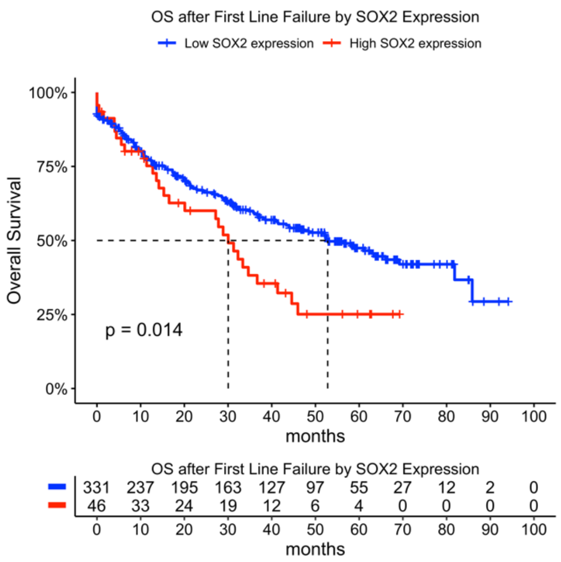

b.

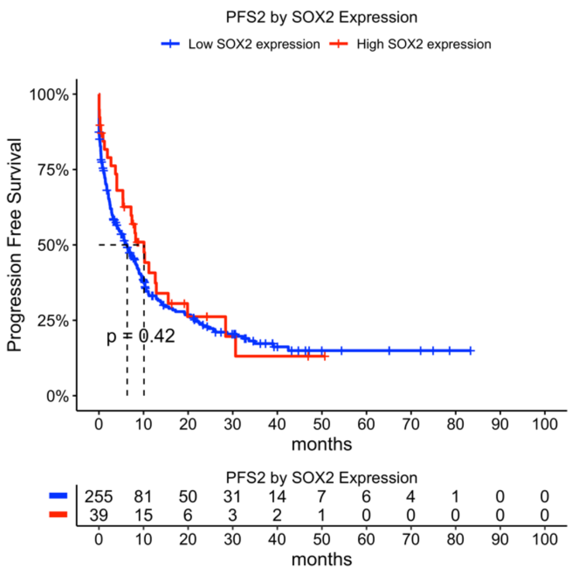

(a) Kaplan-Meier survival curves comparing overall survival after first line therapy failure in 377 patients. OS time is calculated from disease progression to either death or censor. (b) Kaplan-Meier survival curves comparing progression free survival to second line therapy amongst 294 patients who had disease relapse and received second line treatment.

Supplementary Figure 4. Volcano plot of Differential Gene Expression(DGE) between the high SOX2 expression and low SOX2 expression groups.

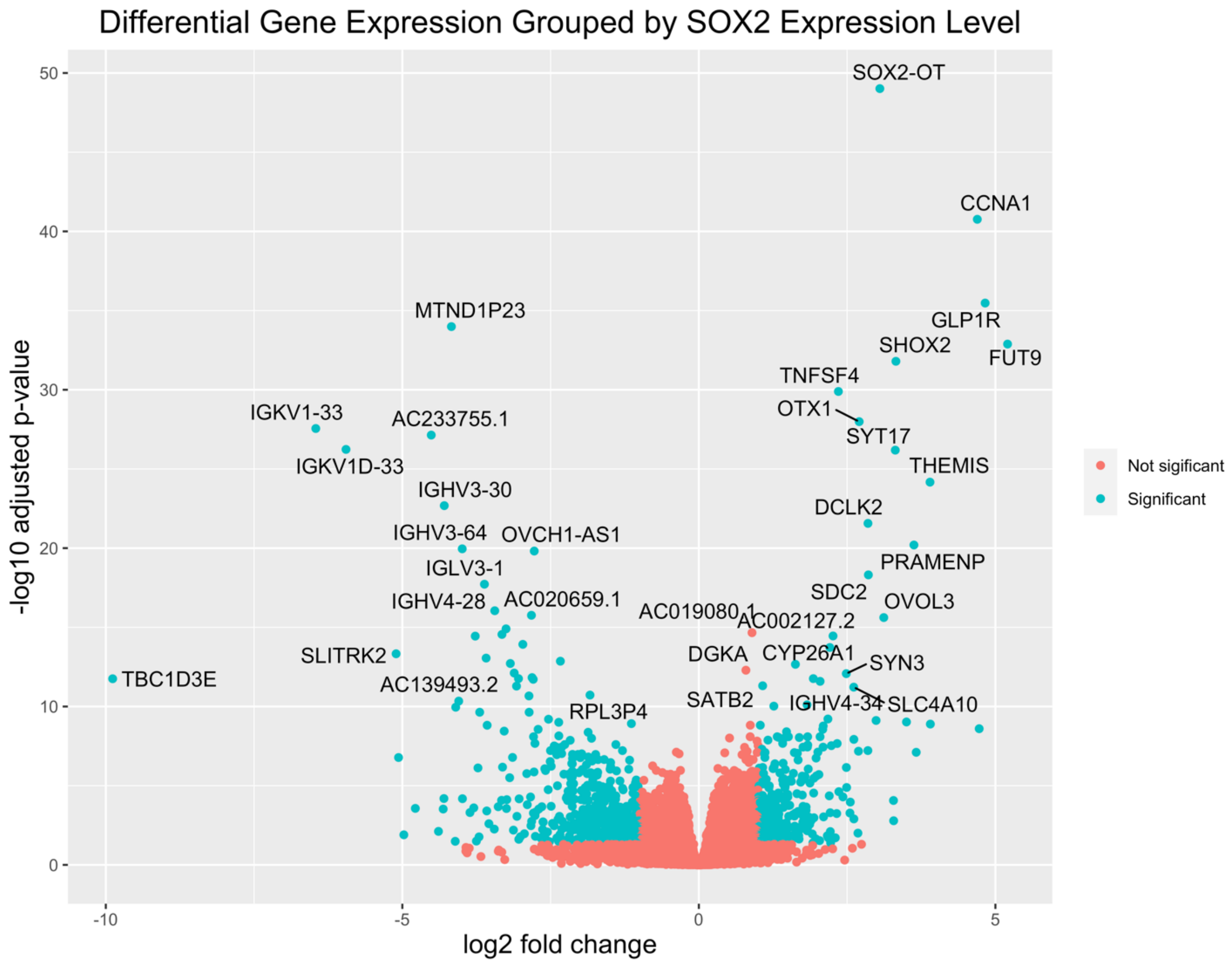

The threshold for significance was a log2 fold change greater than 1 and an adjusted p-value less than 0.05. The green dots represent significantly up-regulated and down-regulated genes.

Supplementary Figure 5. Volcano plot of DGE of stemness genes

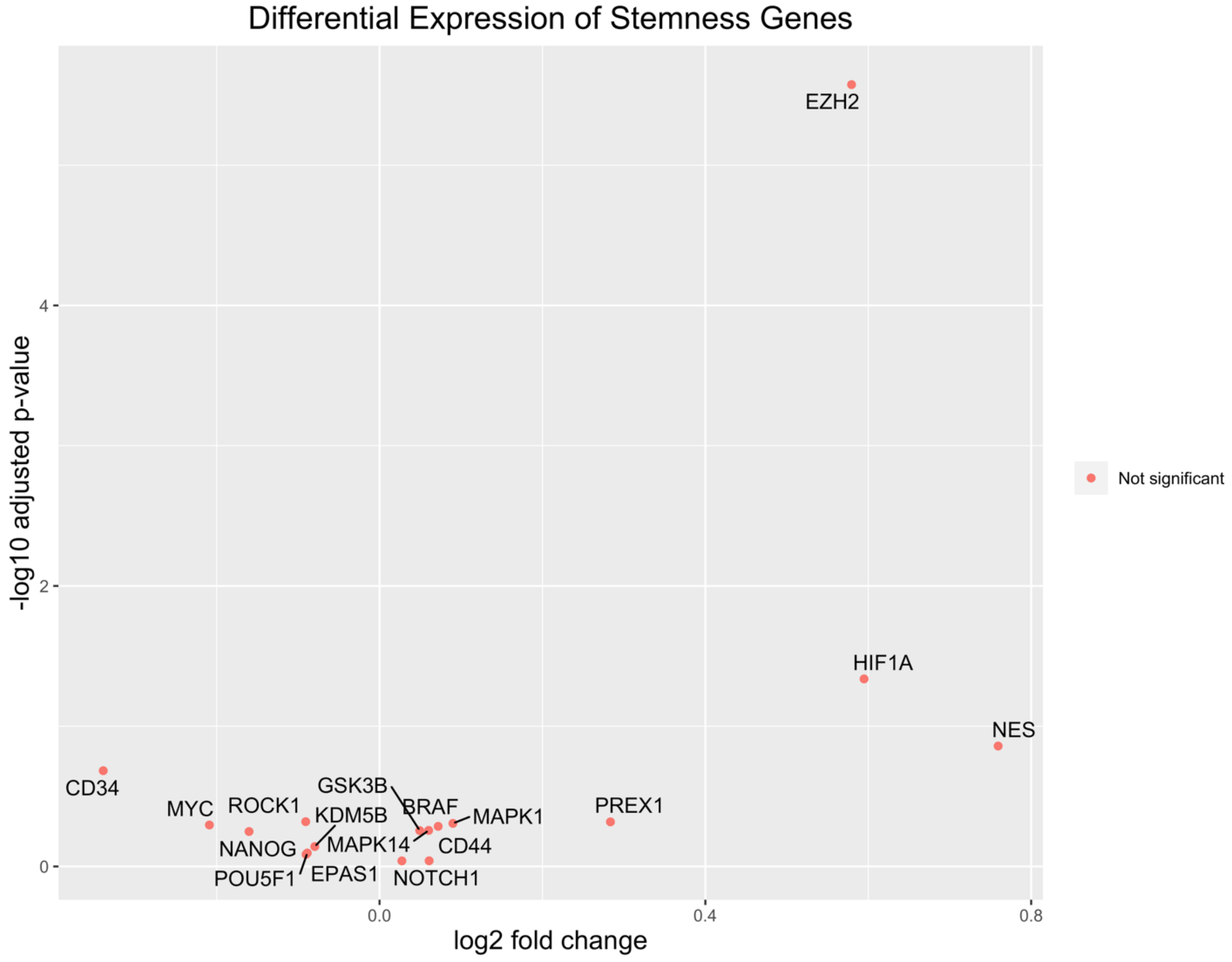

A list of stemness genes were curated and plotted. The threshold for significance was a log2 fold change greater than 1 and an adjusted p-value less than 0.05. Red dots indicate no significant difference in gene expression between the high SOX2 expression group and low SOX2 expression group.

Supplementary Figure 6. Gene Ontology enrichment bubble plots of differentially expressed genes between the high and low SOX2 expression groups.

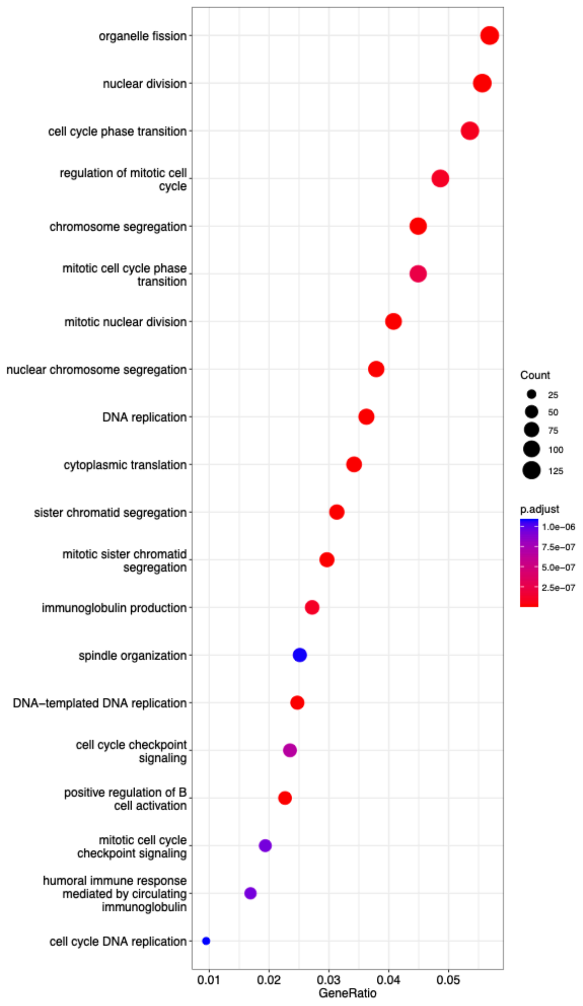

The top 20 enriched KEGG pathways are listed on the vertical axis in a descending order. Gene Ratio, the percentage of total DEGs in the particular GO term, is assigned to the x-axis. The circle's color represents the p value of enrichment significance (red represents higher significance than blue). The size of the circle shows the numbers of the genes enriched in each pathway.
